# Effectiveness of deep learning reconstruction on standard to ultra-low-dose high-definition chest CT images

**DOI:** 10.1007/s11604-023-01470-7

**Published:** 2023-07-27

**Authors:** Nayu Hamabuchi, Yoshiharu Ohno, Hirona Kimata, Yuya Ito, Kenji Fujii, Naruomi Akino, Daisuke Takenaka, Takeshi Yoshikawa, Yuka Oshima, Takahiro Matsuyama, Hiroyuki Nagata, Takahiro Ueda, Hirotaka Ikeda, Yoshiyuki Ozawa, Hiroshi Toyama

**Affiliations:** 1 Department of Radiology, Fujita Health University School of Medicine, 1-98 Dengakugakubo, Kutsukake-cho, Toyoake, Aichi 470-1192 Japan; 2 Department of Diagnostic Radiology, Fujita Health University School of Medicine, Toyoake, Aichi Japan; 3 Joint Research Laboratory of Advanced Medical Imaging, Fujita Health University School of Medicine, Toyoake, Aichi Japan; 4 Canon Medical Systems Corporation, Otawara, Tochigi Japan; 5 Department of Diagnostic Radiology, Hyogo Cancer Center, Akashi, Hyogo Japan

**Keywords:** Lung, CT, Radiation dose, Iterative reconstruction, Deep learning reconstruction

## Abstract

**Purpose:**

Deep learning reconstruction (DLR) has been introduced by major vendors, tested for CT examinations of a variety of organs, and compared with other reconstruction methods. The purpose of this study was to compare the capabilities of DLR for image quality improvement and lung texture evaluation with those of hybrid-type iterative reconstruction (IR) for standard-, reduced- and ultra-low-dose CTs (SDCT, RDCT and ULDCT) obtained with high-definition CT (HDCT) and reconstructed at 0.25-mm, 0.5-mm and 1-mm section thicknesses with 512 × 512 or 1024 × 1024 matrixes for patients with various pulmonary diseases.

**Materials and methods:**

Forty age-, gender- and body mass index-matched patients with various pulmonary diseases underwent SDCT (CT dose index volume <CTDI_vol_>: mean ± standard deviation, 9.0 ± 1.8 mGy), RDCT (CTDI_vol_: 1.7 ± 0.2 mGy) and ULDCT (CTDI_vol_: 0.8 ± 0.1 mGy) at a HDCT. All CT data set were then reconstructed with 512 × 512 or 1024 × 1024 matrixes by means of hybrid-type IR and DLR. SNR of lung parenchyma and probabilities of all lung textures were assessed for each CT data set. SNR and detection performance of each lung texture reconstructed with DLR and hybrid-type IR were then compared by means of paired *t* tests and ROC analyses for all CT data at each section thickness.

**Results:**

Data for each radiation dose showed DLR attained significantly higher SNR than hybrid-type IR for each of the CT data (*p* < 0.0001). On assessments of all findings except consolidation and nodules or masses, areas under the curve (AUCs) for ULDCT with hybrid-type IR for each section thickness (0.91 ≤ AUC ≤ 0.97) were significantly smaller than those with DLR (0.97 ≤ AUC ≤ 1, *p* < 0.05) and the standard protocol (0.98 ≤ AUC ≤ 1, *p* < 0.05).

**Conclusion:**

DLR is potentially more effective for image quality improvement and lung texture evaluation than hybrid-type IR on all radiation dose CTs obtained at HDCT and reconstructed with each section thickness with both matrixes for patients with a variety of pulmonary diseases.

## Introduction

Since multidetector-row CT (MDCT) became widely clinically applied in the mid-2000s, academic and social interests in radiation dose reduction for MDCT examinations without any decline in diagnostic capability have been continuing and led to recommendations for its use to all imaging vendors. Moreover, hybrid-type and model-based iterative reconstruction (IR) methods have been introduced and used for CT examinations and continuously improved since the 2010s [1–7]. In addition, deep learning reconstruction (DLR) has been introduced by some major imaging vendors and tested for CT examinations of a variety of organs as well as compared with other reconstruction methods in both in vivo and in vitro studies [8–13].

During the same periods, the number of detector rows for MDCT has been increased for wider coverage within one rotation as well as reduction in detector collimations. High-definition CTs (HDCTs) with and without a photon counting system have been clinically applied and tested since 2015 [11, 13–24]. One of these HDCTs, an ultra-high-resolution or super-high-resolution CT (UHR-CT or SHR-CT) which is widely available in routine clinical practice, is tested by many investigators using in vitro and in vivo studies [11, 13, 14, 16–20]. This CT system has three different scan modes: normal resolution (NR: 0.5 mm × 80 rows/896 channels), high-resolution (HR: 0.5 mm × 80 rows/1792 channels) and super-high-resolution (SHR: 0.25 mm × 160 rows/1792 channels), and improvements in spatial resolutions for UHR-CT have been reported by several investigators [11, 13, 14, 16–20]. Moreover, UHR-CT makes it possible to use larger matrix sizes such as 1024 or 2048 for certain CT examinations, and it has been suggested this may be useful for some clinical purposes [11, 13, 14, 16–20]. However, one of the drawbacks of UHR-CT might be the relative reduction in signal-to-noise ratio (SNR) and contrast-to-noise ratio (CNR) due to a decrease in the detector collimation size, even when the same radiation dose protocol with standard reconstruction algorithms is used [11, 13, 14, 16–20, 25]. To the best of our knowledge, however, no one has tried reducing the radiation dose for HDCT or evaluated the capability of DLR for image quality improvement on reduced- and ultra-low-dose chest CTs in comparison with clinically applicable IRs for patients with a variety of chest diseases.

We hypothesized that, in comparison with hybrid-type IR, the DLR algorithm makes it possible to improve image noise and reduce radiation dose while maintaining the capability for evaluation of radiological findings for chest reduced- and ultra-low-dose CT examinations of a variety of chest disease patients. The purpose of this study was to compare the capabilities of DLR for image quality improvement and more effective lung texture evaluation with those of hybrid-type IR for standard-, reduced- and ultra-low-dose CTs (SDCT, RDCT and ULDCT, respectively) obtained with high-definition CT (HDCT) and reconstructed at 0.25-mm, 0.5-mm and 1-mm section thicknesses with 512 × 512 or 1024 × 1024 matrixes for patients with a variety of pulmonary diseases.

## Materials and methods

This retrospective study was approved by the Institutional Review Board of Fujita Health University Hospital, and is compliant with the Health Insurance Portability and Accountability Act. Written informed consent was waived. This study was technically or financially supported by Canon Medical Systems Corporation, the Smoking Research Foundation and a Grant-in-Aid for Scientific Research from the Japanese Ministry of Education, Culture, Sports, Science and Technology (JSTS KAKEN No. 20K08037). In addition, this study was partly supported by J-QIBA in Japan Radiological Society. Four of the authors are employees of Canon Medical Systems Corporation (H.K., Y.I., K.F. and N.A.) but did not have control over any of the data used in this study.

### Subjects

**Fig. 1:**
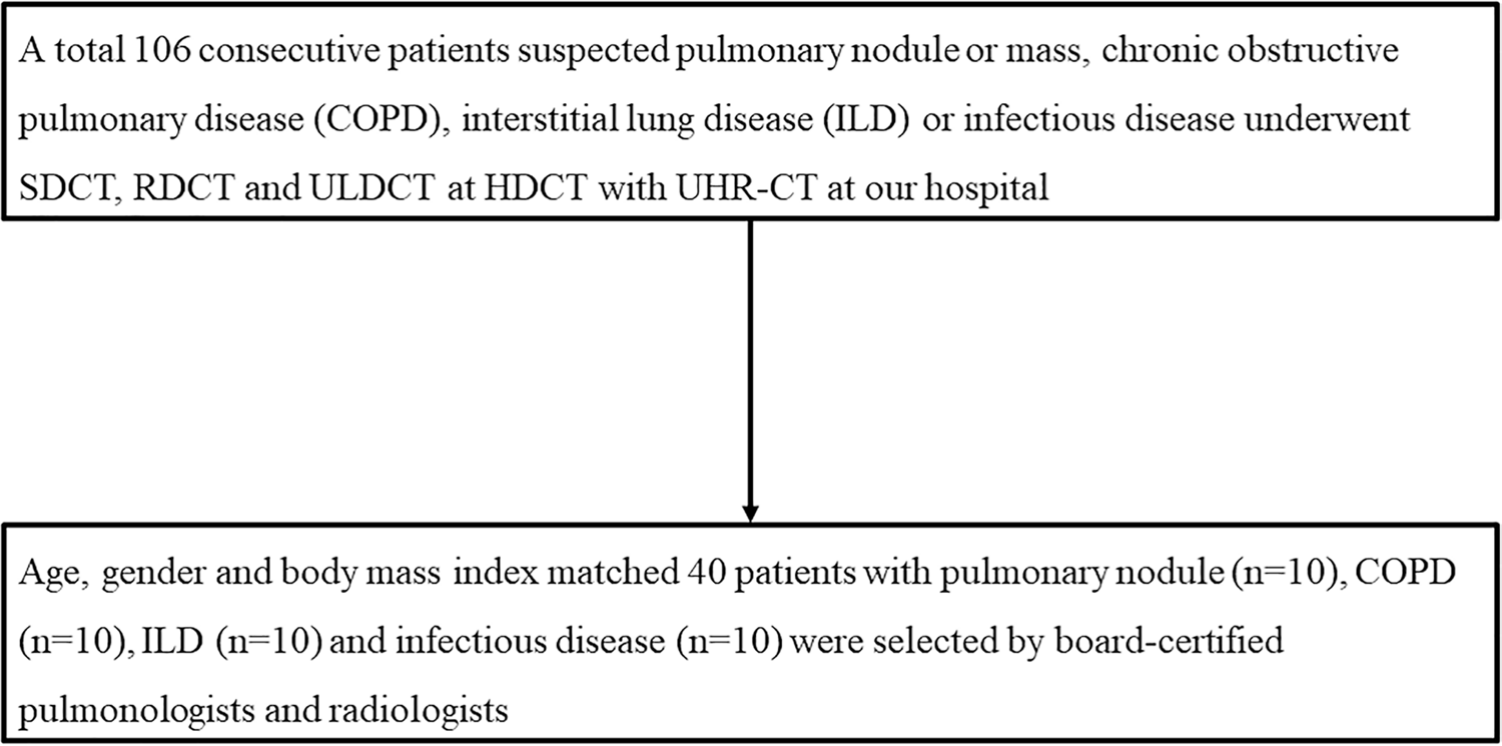
Patient selection flowchart. Final study population was selected from the original cohort

A total of 106 consecutive patients with suspected presence of pulmonary nodules or mass, chronic obstructive pulmonary disease (COPD), interstitial lung disease (ILD) or infectious disease underwent SDCT, RDCT and ULDCT with a HDCT at our hospital between December 2020 and May 2021. Next, 40 age-, gender- and body mass index-matched patients as having pulmonary nodules (*n* = 10), COPD (*n* = 10), ILD (*n* = 10) or infectious disease (*n* = 10) were selected by board-certified pulmonologists and radiologists with more than 10 years’ experience who did not take part in this study. This study group of 40 patients (mean age, 66 years; age range, 41–86 years) comprised 24 males (mean age, 66 years; age range, 43–85 years) and 16 females (mean age, 66 years; age range, 41–86 years) with ten cases of COPD, six of pulmonary tuberculosis, five with invasive adenocarcinomas, four with idiopathic pulmonary fibrosis (IPF), three each with minimally invasive adenocarcinomas (MIAs) and nontuberculous mycobacteria (NTM) infections, two each with adenocarcinoma in situ (AIS) and pulmonary asbestosis, and one each with non-specific interstitial pneumonia (NSIP), progressive systemic sclerosis (PSS), rheumatoid arthritis and a mixed connective tissue disorder and coronavirus disease 2019 (COVID-19). Moreover, eight out of ten non-small cell lung cancer (NSCLC) cases had emphysema (*n* = 5), interstitial lung abnormality (*n* = 4), post-infectious changes (*n* = 3) due to NTM (*n* = 2) or pulmonary tuberculosis (*n* = 1) or pulmonary asbestosis with or without asbestos-related pleural disease (*n* = 3). In addition, seven out of ten infectious disease patients had pulmonary emphysema (*n* = 5) or interstitial lung abnormality (*n* = 4). Furthermore, five out of ten COPD patients and four out of ten ILD patients had pulmonary nodules, which were not histologically confirmed and followed up by CT every 6 months. Some of each selected group cases had a few additional lung abnormalities. The patient selection flowchart is shown in Fig. 1.

### HDCT protocol

All unenhanced chest HDCT examinations were performed with 160-detector row CT scanners (Aquilion Precision; Canon Medical Systems, Otawara, Tochigi, Japan). HDCT images of the whole chest were obtained with a single breath-hold using the following tube currents: for SDCT, it was modified by an automatic exposure control (AEC) system (CTDI_vol_: mean ± standard deviation, 9.0 ± 1.8 mGy; image SD = 15); for RDCT, it was 30 mA (CTDI_vol_: 1.7 ± 0.2 mGy); and for ULDCT, it was 15 mA (CTDI_vol_: 0.8 ± 0.1 mGy). Three consecutive helical acquisitions were performed for each patient with helical scans of the same length and the same field of view to obtain three volume data sets of the whole chest. Other scan parameters were the same for each CT protocol: peak tube voltage, 120 kV; gantry speed, 0.5 s/rotation; detector collimation, 0.25 mm × 160 rows/1792 channels (SHR mode); beam pitch, 0.569. We therefore had three raw-data files of the same size for all 40 patients. The SDCT, RDCT and ULDCT data sets were then reconstructed as 0.25-mm-thick sections with 512 and 1024 matrices and as 0.5- and 1-mm-thick sections with a 512 matrix reconstructed with hybrid-type IR (adaptive iterative dose reduction using 3D processing: AIDR 3D, Canon Medical Systems) as the standard level (i.e., AIDR 3D STD) by using a lung kernel (FC52, Canon Medical Systems) and DLR (Advanced Intelligent Clear-IQ Engine: AiCE, Canon Medical Systems) for lung as the standard level (i.e., AiCE Lung STD) at the lung window setting. Details of the CT protocol are shown in Table 1.

**Table 1:** Details of CT protocol

	SDCT	RDCT	ULDCT
CT System	160-detector row CT (Aquilion Precision: Canon Medical Systems)		
Scan mode	SHR		
Detector collimation	0.25 mm × 160 rows/1792 channels		
Tube current (mA)	Automatic exposure control at image SD of 15	30	15
Tube voltage (kVp)	120		
Beam pitch	0.569		
Gantry speed (s/rotation)	0.5		
Section thickness (mm)	0.25, 0.5 and 1		
Matrix	512 × 512 matrix for all section thicknesses and 1024 × 1024 matrix for 0.25-mm section thickness		
Reconstruction method and kernel	Hybrid-type iterative reconstruction (AIDR 3D: Canon Medical Systems) using a lung kernel (FC52: Canon Medical Systems)		
	Deep learning reconstruction (AiCE Lung: Canon Medical Systems)		
Radiation dose (CTDI_vol_: mGy) (mean ± standard deviation)	9.0 ± 1.8	1.7 ± 0.2	0.8 ± 0.1

*SDCT* standard-dose CT, *RDCT* reduced-dose CT, *ULDCT* ultra-low-dose CT, *SHR* super high-resolution

### Image analysis

All HDCT images were randomized for an independent review by a senior fellow (N.H.) and a board-certified chest radiologist (Y.O.) with 28 years of experience. For this study, all HDCT data sets at lung window setting (level: − 550 HU, width: 1600 HU) were randomly interpreted by using a picture archiving and communication system (RapidCore; Canon Medical Systems) with the reviewers having no access to information about the technical parameters. All CT image evaluations were performed by two radiologists at different times, days and reading rooms.

#### Quantitative assessment of image noise

Images obtained at the level of the aortic arch, carina, and lung bases were used for quantitative analysis of image quality. First, circular regions of interest (ROIs) 10 mm in diameter were then placed on the lung window setting at the tracheal lumen and bilateral lung parenchyma on SDCT data, and all ROIs copied to other CT data. Lung parenchyma CT values and image noise within both lungs were determined for each patient as the mean CT value and standard deviation (SD) within the ROI in both lungs at different levels [1–5, 11]. Image noise within the trachea was also determined as the standard deviation (SD) of the CT value within the ROI at the trachea [1–5, 11]. Therefore, a total of 168 ROI measurements were performed (3 radiation doses × 2 reconstruction methods × 4 different section thickness and reconstruction matrix × 7 ROIs) to determine all indexes as well as the signal-to-noise ratio (SNR) for each patient. SNR is determined with the following formula [1–5, 11]:

1 $${\text{SNR of lung}} = {\text{mean CT value for lung parenchyma}}/{\text{SD at trachea}}$$

#### Qualitative assessment of image quality

To compare image noise reduction capability of each CT series with that of SDCT, a 5-point visual scoring system for evaluation of overall image quality was adopted: 1, non-diagnostic; 2, poor; 3, acceptable; 4, good; and 5, excellent. Moreover, artifact, which was determined as sum of noise, streak artifact, blurring of the border between the lung and chest wall, was also assessed by a 5-point visual scoring system as follows: 1, absent; 2, probably absent; 3, equivocal; 4, probably present; and 5, present. These systems were used to evaluate image quality at the level of the lung apices, aortic arch, carina, left atrium, and lung bases on all CT images. When evaluated overall image quality, suitability of a set of images was defined as a consistent score of 3 (acceptable) or more for all five levels in all patients (a total of 9600 lung areas: 40 patients × 3 radiation doses × 2 reconstruction methods × 4 different section thicknesses and reconstruction matrixes × 5 levels × 2 lungs). When assessed artifact in each patient, suitability of a set of images was defined as a consistent score of 3 (equivocal) or less for all five levels (a total of 9600 lung areas: 40 patients × 3 radiation doses × 2 reconstruction methods × 4 different section thicknesses and reconstruction matrixes × 5 levels × 2 lungs).

To compare the lung texture evaluation capability of all CT protocols reconstructed with DLR and hybrid-type IR with the same section thickness and reconstruction matrix, a 5-point visual scoring system, 1, absent; 2, probably absent; 3, equivocal; 4, probably present; and 5, present, was used to determine the presence of (a) emphysema, (b) ground glass opacity (GGO), (c) reticulation, (d) nodular lesion, (e) consolidation, (f) honeycombing and (g) nodule or mass based on the glossary terms for thoracic imaging published by the Fleischner Society [26] for all CT protocols with the above-mentioned rule (a total of 9600 lung areas: 40 patients × 3 radiation doses × 2 reconstruction methods × 4 different section thicknesses and reconstruction matrixes × 5 levels, which were same level for qualitative image quality evaluation, × 2 lungs).

Moreover, reference standards were determined on thin-section SDCTs reconstructed with hybrid-type IR for every patient and were reviewed and established by the consensus of two board certified radiologists with more than 21 years’ experience (T.Y. and Yo.O.).

### Statistical analysis

To determine the capability of DLR compared with that of hybrid-type IR for improving quantitative image quality using each of the HDCT protocols, image noise and SNR within the DLR processed lung parenchyma were compared by using the paired t test with those within the hybrid-type IR processed lung parenchyma at each section thickness reconstructed with a 512 × 512 or 1024 × 1024 matrix.

To compare image quality and artifact reduction capability between DLR and hybrid-type IR on all CT protocols at same section thickness, overall image quality and artifact were compared by Wilcoxon’s signed rank test between DLR and hybrid-type IR at each radiation dose and between SDCT with hybrid-type IR and others. Moreover, kappa statistics were used to determine interobserver agreements for overall image analysis and artifact evaluations obtained with each CT protocol.

Receiver operating characteristic (ROC) analysis was used to compare detection accuracy of all CT protocols for each lung texture at the same section thickness. For this statistical analysis, SDCT reconstructed with hybrid-type IR at 1-mm section thickness was defined as the standard protocol in this study.

In addition, kappa statistics were used to evaluate interobserver agreements for all radiological finding assessments obtained with each CT protocol.

All agreements were considered slight for *κ* < 0.21, fair for *κ* = 0.21–0.40, moderate for *κ* = 0.41–0.60, substantial for *κ* = 0.61–0.80, and almost perfect for *κ* = 0.81–1.00 [27].

A *p* value less than 0.05 was considered sufficiently significant for statistical analyses.

## Results

**Fig. 2:**
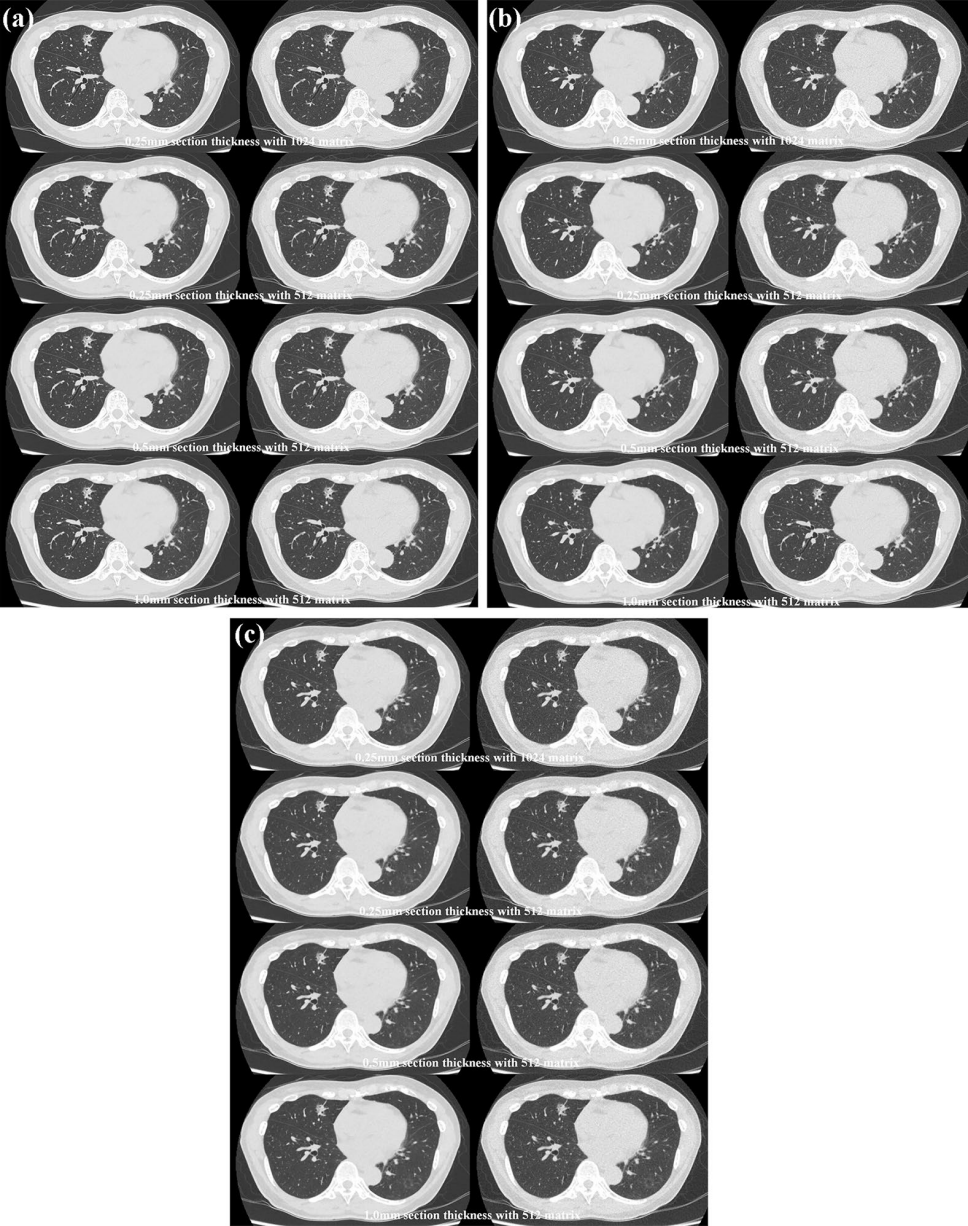
Seventy-year-old female with invasive adenocarcinoma in the right middle lobe. **A** (L: SDCT reconstructed with DLR; R: SDCT with hybrid-type IR) For each SDCT data set, there was less image noise for each SDCT reconstructed with DLR than when reconstructed with hybrid-type IR. All SDCTs clearly showed a partly solid nodule with a 12-mm-long axis, a pleural tag and an enlarged airway in the middle lobe. 0.25-mm section thickness with 1024 matrix with DLR and hybrid-type IR were scored as 5 and 5, and 0.25-mm section thickness with 512 matrix with DLR and hybrid-type IR were also scored as 5 and 5. 0.5-mm section thickness with 512 matrix with DLR and hybrid-type IR were scored as 5 and 5, and 1-mm section thickness with 512 matrix with DLR and hybrid-type IR were also scored as 5 and 5. **B** (L: RDCT reconstructed with DLR; R: RDCT reconstructed with hybrid-type IR) Image noise of RDCT reconstructed with DLR was less than of that reconstructed with hybrid-type IR. All RDCTs clearly demonstrated the presence of a partly solid nodule in the middle lobe, a pleural tag and an enlarged airway. No differences in radiological findings were observed between any RDCT and either of the two SDCTs with the same section thickness and matrix size. 0.25-mm section thickness with 1024 matrix with DLR and hybrid-type IR were scored as 5 and 5, and 0.25-mm section thickness with 512 matrix with DLR and hybrid-type IR were also scored as 5 and 5. 0.5-mm section thickness with 512 matrix with DLR and hybrid-type IR were scored as 5 and 5, and 1-mm section thickness with 512 matrix with DLR and hybrid-type IR were also scored as 5 and 5. **C** (L: ULDCT reconstructed with DLR; R: ULDCT reconstructed with hybrid-type IR) For each ULDCT data set, image noise of each ULDCT reconstructed with DLR was lower than of one reconstructed with hybrid-type IR. All ULDCTs showed a partly solid nodule, a pleural tag and an enlarged airway in the middle lobe, and no differences were observed between radiological findings on any ULDCT, any of the SDCTs and RDCTs with the same section thickness and reconstruction matrix. 0.25-mm section thickness with 1024 matrix with DLR and hybrid-type IR were scored as 5 and 5, and 0.25-mm section thickness with 512 matrix with DLR and hybrid-type IR were also scored as 5 and 5. 0.5-mm section thickness with 512 matrix with DLR and hybrid-type IR were scored as 5 and 5, and 1-mm section thickness with 512 matrix with DLR and hybrid-type IR were also scored as 5 and 5

**Fig. 3:**
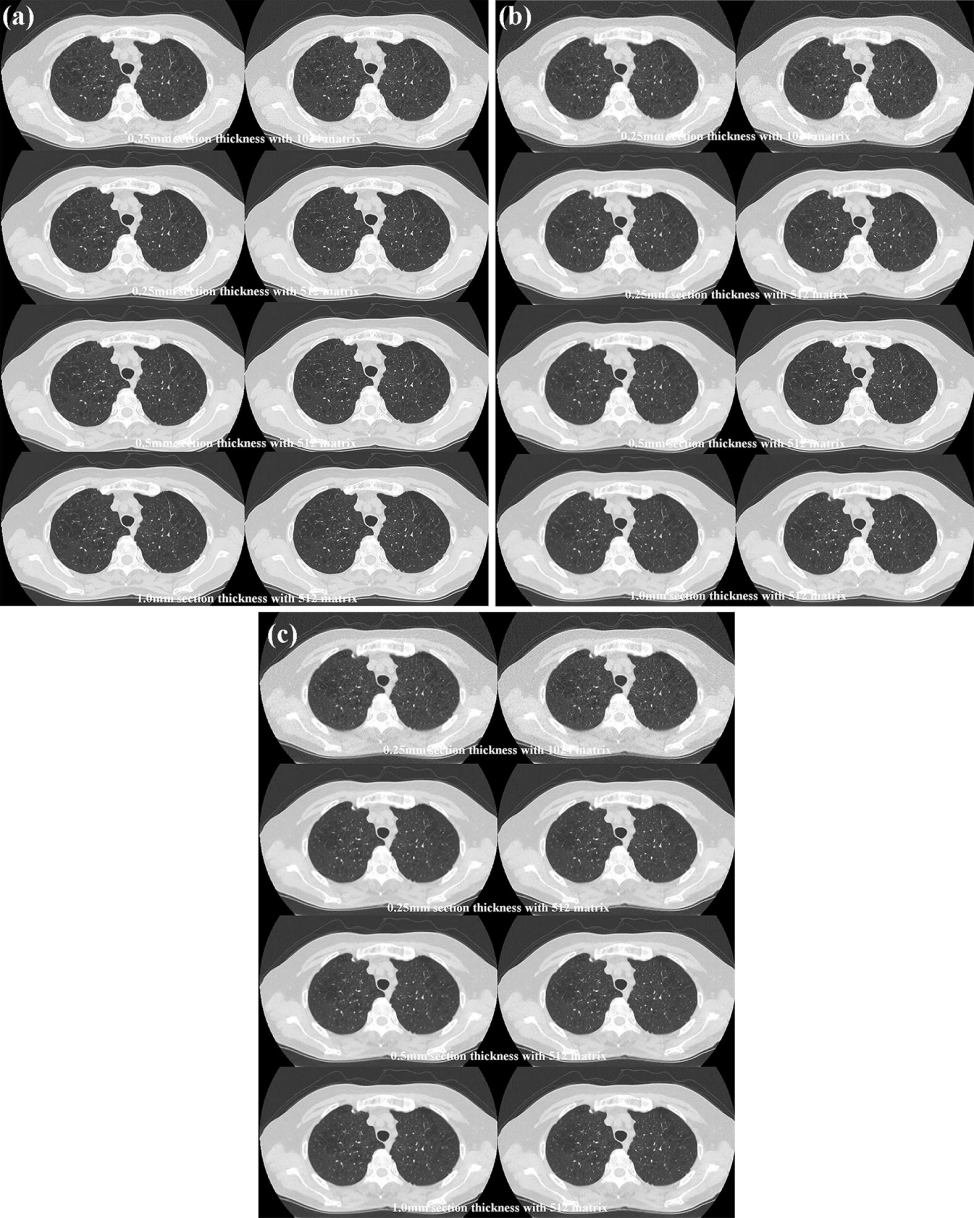
Sixty-eight-year-old male with pulmonary emphysema in both upper lobes. **A** (L: SDCT reconstructed with DLR; R: SDCT with hybrid-type IR) For each SDCT data set, there was less image noise on each SDCT reconstructed with DLR than when reconstructed with hybrid-type IR. All SDCTs clearly showed low attenuation areas in both upper lobes, which allowed a diagnosis of centrilobular emphysema. 0.25-mm section thickness with 1024 matrix with DLR and hybrid-type IR were scored as 5 and 5, and 0.25-mm section thickness with 512 matrix with DLR and hybrid-type IR were also scored as 5 and 5. 0.5-mm section thickness with 512 matrix with DLR and hybrid-type IR were scored as 5 and 5, and 1-mm section thickness with 512 matrix with DLR and hybrid-type IR were also scored as 5 and 5. **B** (L: RDCT reconstructed with DLR; R: RDCT reconstructed with hybrid-type IR) Image noise of RDCT reconstructed with DLR was less than when reconstructed with hybrid-type IR. All RDCTs also clearly showed low attenuation areas in both upper lobes and demonstrated there was no difference between any two SDCTs with the same section thickness and matrix size. 0.25-mm section thickness with 1024 matrix with DLR and hybrid-type IR were scored as 5 and 4, and 0.25-mm section thickness with 512 matrix with DLR and hybrid-type IR were also scored as 5 and 4. 0.5-mm section thickness with 512 matrix with DLR and hybrid-type IR were scored as 5 and 4, and 1-mm section thickness with 512 matrix with DLR and hybrid-type IR were also scored as 5 and 4. **C** (L: ULDCT with DLR; R: ULDCT with hybrid-type IR) Data for each ULDCT showed that image noise of each ULDCT reconstructed with DLR was lower than when reconstructed with hybrid-type IR. All ULDCTs demonstrated the presence of low attenuation areas in both upper lobes, although visualization of each low attenuation area was less effective than that on both SDCTs and RDCTs with the same section thickness and reconstruction matrix. 0.25-mm section thickness with 1024 matrix with DLR and hybrid-type IR were scored as 4 and 3, and 0.25-mm section thickness with 512 matrix with DLR and hybrid-type IR were also scored as 4 and 3. 0.5-mm section thickness with 512 matrix with DLR and hybrid-type IR were scored as 4 and 3, and 1-mm section thickness with 512 matrix with DLR and hybrid-type IR were also scored as 4 and 3

**Fig. 4:**
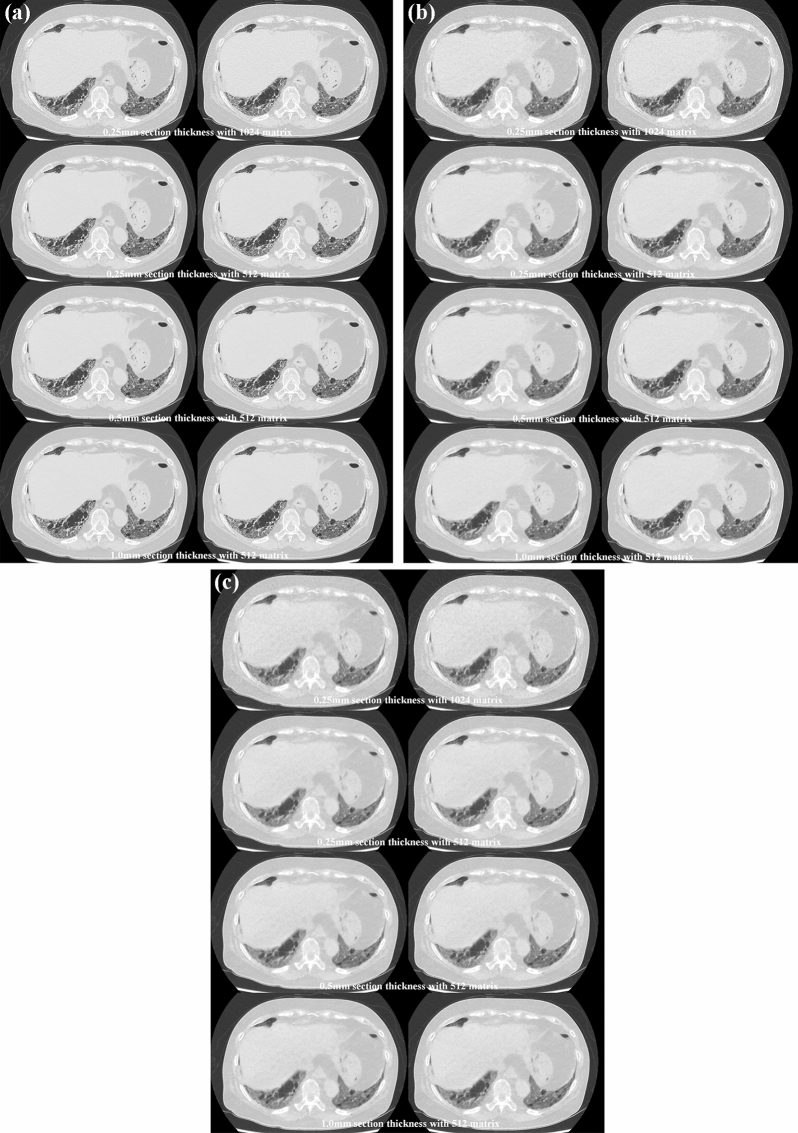
Sixty-five-year-old female with interstitial lung disease due to mixed connective tissue disease (MCTD). **A** (L: SDCT reconstructed with DLR; R: SDCT reconstructed with hybrid-type IR) For each SDCT data set, image noise on each SDCT reconstructed with DLR was lower than when reconstructed with hybrid-type IR. All SDCTs clearly demonstrated reticulation in the right lower lobe and GGOs in both lower lobes and thus allowed a diagnosis of interstitial lung disease due to MCTD. 0.25-mm section thickness with 1024 matrix with DLR and hybrid-type IR were scored as 5 and 5, and 0.25-mm section thickness with 512 matrix with DLR and hybrid-type IR were also scored as 5 and 5. 0.5-mm section thickness with 512 matrix with DLR and hybrid-type IR were scored as 5 and 5, and 1-mm section thickness with 512 matrix with DLR and hybrid-type IR were also scored as 5 and 5. **B** (L: RDCT reconstructed with DLR; R: RDCT reconstructed with hybrid-type IR) Image noise on RDCT reconstructed with DLR was less than when reconstructed with hybrid-type IR. All RDCTs also demonstrated the presence of reticulation in the right lower lobe and GGOs in both lower lobes. Moreover, visualization of reticulation in the right lower lobe was less effective on RDCT reconstructed with DLR and hybrid-type IR than on any SDCT with the same section thickness and matrix size. However, RDCT with DLR or hybrid-type IR allowed a diagnosis of this patient as having interstitial lung disease due to MCTD. 0.25-mm section thickness with 1024 matrix with DLR and hybrid-type IR were scored as 4 and 3, and 0.25-mm section thickness with 512 matrix with DLR and hybrid-type IR were also scored as 4 and 3. 0.5-mm section thickness with 512 matrix with DLR and hybrid-type IR were scored as 4 and 3, and 1-mm section thickness with 512 matrix with DLR and hybrid-type IR were also scored as 4 and 3. **C** (L: ULDCT reconstructed with DLR; R: ULDCT reconstructed with hybrid-type IR) Data for ULDCT demonstrated that image noise on each ULDCT reconstructed with DLR was lower than when reconstructed with hybrid-type IR. All ULDCTs demonstrated GGOs in both lower lobes, while reticulation in the right lower lobe was markedly less and visualized as GGOs. A comparison of SDCT and RDCT reconstructed with each method at the same section thickness and matrix size and of each ULDCT showed that interstitial abnormalities could not be clearly demonstrated, although a diagnosis of interstitial lung disease due to MCTD could be established. 0.25-mm section thickness with 1024 matrix with DLR and hybrid-type IR were scored as 3 and 2, and 0.25-mm section thickness with 512 matrix with DLR and hybrid-type IR were also scored as 3 and 2. 0.5-mm section thickness with 512 matrix with DLR and hybrid-type IR were scored as 3 and 2, and 1-mm section thickness with 512 matrix with DLR and hybrid-type IR were also scored as 3 and 2

Representative cases of SDCT, RDCT and ULDCT reconstructed at each section thickness with 512 × 512 or 1024 × 1024 matrixes reconstructed with hybrid-type IR and DLR are shown in Figs. 2, 3 and 4.

Results of a comparison of quantitative image quality indexes between DLR and hybrid-type IR for each CT protocol are shown in Table 2. For each of the radiation dose data, DLR showed significantly lower image noise and higher SNR of lung parenchyma than hybrid-type IR on CTs at each section thickness and 512 × 512 or 1024 × 1024 matrixes (image noise: *p* < 0.0001, SNR: *p* < 0.0001).

**Table 2:** Comparison of quantitative image quality indexes for DLR and hybrid-type IR for each CT protocol

Section thickness (mm)	Reconstruction matrix	HDCT protocols	Reconstruction method	Image noise (HU)		SNR	
				(mean ± standard deviation)	*p* value	(mean ± standard deviation)	*p* value
1	512 × 512	SDCT	DLR	29.9 ± 14.8	< 0.0001	82.6 ± 8.1	< 0.0001
			Hybrid-type IR	35.2 ± 12.2		35.4 ± 4.1	
		RDCT	DLR	37.4 ± 24.2	< 0.0001	62.2 ± 11.1	< 0.0001
			Hybrid-type IR	46.0 ± 19.1		26.9 ± 2.7	
		ULDCT	DLR	35.9 ± 30.0	< 0.0001	54.6 ± 7.1	< 0.0001
			Hybrid-type IR	46.8 ± 25.3		27.5 ± 2.7	
0.5	512 × 512	SDCT	DLR	32.5 ± 15.4	< 0.0001	71.4 ± 6.1	< 0.0001
			Hybrid-type IR	38.7 ± 12.3		30.9 ± 3.5	
		RDCT	DLR	41.1 ± 25.8	< 0.0001	56.2 ± 10.5	< 0.0001
			Hybrid-type IR	49.6 ± 19.9		24.4 ± 4.1	
		ULDCT	DLR	37.8 ± 31.4	< 0.0001	50.8 ± 6.0	< 0.0001
			Hybrid-type IR	49.9 ± 26.2		25.8 ± 2.6	
0.25	512 × 512	SDCT	DLR	37.5 ± 14.4	< 0.0001	55.1 ± 11.9	< 0.0001
			Hybrid-type IR	45.7 ± 11.8		24.9 ± 4.9	
		RDCT	DLR	39.8 ± 20.5	< 0.0001	48.3 ± 9.3	< 0.0001
			Hybrid-type IR	52.4 ± 15.0		21.8 ± 2.6	
		ULDCT	DLR	37.4 ± 31.4	< 0.0001	45.9 ± 5.6	< 0.0001
			Hybrid-type IR	51.6 ± 26.6		23.9 ± 2.9	
0.25	1024 × 1024	SDCT	DLR	41.7 ± 15.3	< 0.0001	43.8 ± 1.8	< 0.0001
			Hybrid-type IR	51.7 ± 11.3		20.6 ± 1.6	
		RDCT	DLR	46.1 ± 26.4	< 0.0001	42.7 ± 2.5	< 0.0001
			Hybrid-type IR	58.6 ± 19.4		20.0 ± 1.6	
		ULDCT	DLR	41.5 ± 31.7	< 0.0001	42.7 ± 3.2	< 0.0001
			Hybrid-type IR	56.3 ± 25.8		22.6 ± 2.6	

*SDCT* standard-dose CT, *RDCT* reduced-dose CT, *ULDCT* ultra-low-dose CT, *IR* iterative reconstruction, *DLR* deep learning reconstruction

Interobserver agreements for overall image quality and artifact evaluations are shown in Table 3. For all CT data sets, interobserver agreements for overall image quality (0.67 ≤ *κ* ≤ 1, *p* < 0.0001) and artifacts (0.68 ≤ *κ* ≤ 1, *p* < 0.0001) were determined as substantial or almost perfect.

**Table 3:** Interobserver agreements for overall image quality and artifact evaluations using all CT protocols

Section thickness (mm)	Reconstruction matrix	HDCT protocols	Reconstruction method	Image quality		Artifact	
				*κ*	*p* value	*κ*	*p* value
1	512 × 512	SDCT	DLR	1	< 0.0001	1	< 0.0001
			Hybrid-type IR	1	< 0.0001	1	< 0.0001
		RDCT	DLR	0.81	< 0.0001	0.79	< 0.0001
			Hybrid-type IR	0.67	< 0.0001	0.68	< 0.0001
		ULDCT	DLR	0.74	< 0.0001	0.72	< 0.0001
			Hybrid-type IR	0.69	< 0.0001	0.68	< 0.0001
0.5	512 × 512	SDCT	DLR	1	< 0.0001	1	< 0.0001
			Hybrid-type IR	0.96	< 0.0001	0.95	< 0.0001
		RDCT	DLR	0.9	< 0.0001	0.91	< 0.0001
			Hybrid-type IR	0.89	< 0.0001	0.9	< 0.0001
		ULDCT	DLR	0.91	< 0.0001	0.91	< 0.0001
			Hybrid-type IR	0.8	< 0.0001	0.79	< 0.0001
0.25	512 × 512	SDCT	DLR	1	< 0.0001	1	< 0.0001
			Hybrid-type IR	1	< 0.0001	1	< 0.0001
		RDCT	DLR	0.87	< 0.0001	0.87	< 0.0001
			Hybrid-type IR	0.77	< 0.0001	0.76	< 0.0001
		ULDCT	DLR	0.89	< 0.0001	0.89	< 0.0001
			Hybrid-type IR	0.8	< 0.0001	0.8	< 0.0001
0.25	1024 × 1024	SDCT	DLR	0.89	< 0.0001	0.89	< 0.0001
			Hybrid-type IR	1	< 0.0001	1	< 0.0001
		RDCT	DLR	0.85	< 0.0001	0.84	< 0.0001
			Hybrid-type IR	0.79	< 0.0001	0.79	< 0.0001
		ULDCT	DLR	0.89	< 0.0001	0.89	< 0.0001
			Hybrid-type IR	0.78	< 0.0001	0.78	< 0.0001

*SDCT* standard-dose CT, *RDCT* reduced-dose CT, *ULDCT* ultra-low-dose CT, *IR* iterative reconstruction, *DLR* deep learning reconstruction

A comparison of overall image quality and artifacts between DLR and hybrid-type IR for all CT protocols at the same section thickness is shown in Table 4. On CTs with a section thickness of 1 mm, 0.5 mm and 0.25 mm and a 512 × 512 matrix and 0.25-mm section thickness and a 1024 × 1024 matrix, overall image quality and artifacts of DLR showed significant improvements compared with those of hybrid-type IR at each radiation dose (*p* < 0.0001). Moreover, overall image quality and the number of artifacts of RDCT and ULDCT reconstructed with DLR or hybrid-type IR were significantly worse than those for SDCT reconstructed with hybrid-type IR (*p* < 0.0001). In addition, artifacts for RDCT reconstructed with DLR were significantly more numerous than those for SDCT reconstructed with hybrid-type IR (*p* < 0.05).

**Table 4:** Comparison of overall image quality and artifacts for DLR and hybrid-type IR for all CT protocols at a given section thickness

Section thickness (mm)	Reconstruction matrix	HDCT protocols	Reconstruction method	Image quality		Artifacts	
				Median	Interquartile range (IQR)	Median	Interquartile range (IQR)
1	512 × 512	SDCT	DLR	5	5–5	1	1–1
			Hybrid-type IR	5*	5–5	1*	1–1
		RDCT	DLR	5	5–5	1^+^	1–1
			Hybrid-type IR	4*^, +^	4–4	2*^, +^	2–3
		ULDCT	DLR	4^+^	4–5	1^, +^	1–2
			Hybrid-type IR	3*,^+^	3–3	3*^, +^	2–3
0.5	512 × 512	SDCT	DLR	5	5–5	1	1–1
			Hybrid-type IR	5*	5–5	1*	1–1
		RDCT	DLR	5	5–5	1^+^	1–1
			Hybrid-type IR	4*^, +^	4–4	2*^, +^	2–2
		ULDCT	DLR	5^+^	4–5	1^, +^	1–2
			Hybrid-type IR	3*^, +^	3–3	3*^, +^	2–3
0.25	512 × 512	SDCT	DLR	5	5–5	1	1–1
			Hybrid-type IR	5*	5–5	1*	1–1
		RDCT	DLR	5	5–5	1^+^	1–1
			Hybrid-type IR	4*^, +^	4–4	2*^, +^	2–2
		ULDCT	DLR	5^+^	4–5	1^, +^	1–2
			Hybrid-type IR	3*^, +^	3–3.75	3*^, +^	2.25–3
0.25	1024 × 1024	SDCT	DLR	5	5–5	1	1–1
			Hybrid-type IR	5*	5–5	1*	1–1
		RDCT	DLR	5	5–5	1^+^	1–1
			Hybrid-type IR	4*^, +^	3–4	2*^, +^	2–3
		ULDCT	DLR	5^+^	4–5	1^, +^	1–2
			Hybrid-type IR	3*^, +^	2–3	3*^, +^	3–4

*SDCT* standard-dose CT, *RDCT* reduced-dose CT, *ULDCT* ultra-low-dose CT, *IR* iterative reconstruction, *DLR* deep learning reconstruction

*Significant difference with CT obtained at the same radiation dose and reconstructed with DLR for the same section thickness (*p* < 0.05)

^+^Significant difference with standard-dose CT reconstructed with hybrid-type IR at each section thickness (*p* < 0.05)

Interobserver agreements for all lung texture assessments are shown in Table 5. Interobserver agreements for each lung radiological finding were determined to be substantial or almost perfect for all CT data sets (0.72 ≤ *κ* ≤ 0.93, *p* < 0.0001).

**Table 5:** Interobserver agreements for all lung texture assessments using all CT protocols

Section thickness (mm)	Reconstruction matrix	HDCT protocols	Reconstruction method	Emphysema		GGO		Reticulation		Nodular Lesion		Consolidation		Honeycombing		Nodule or Mass	
				*κ*	*p* value	*κ*	*p* value	*κ*	*p* value	*κ*	*p* value	*κ*	*p* value	*κ*	*p* value	*κ*	*p* value
1	512 × 512	SDCT	DLR	0.84	< 0.0001	0.85	< 0.0001	0.88	< 0.0001	0.84	< 0.0001	0.92	< 0.0001	0.86	< 0.0001	0.89	< 0.0001
			Hybrid-type IR	0.85	< 0.0001	0.86	< 0.0001	0.89	< 0.0001	0.85	< 0.0001	0.93	< 0.0001	0.87	< 0.0001	0.90	< 0.0001
		RDCT	DLR	0.82	< 0.0001	0.83	< 0.0001	0.86	< 0.0001	0.83	< 0.0001	0.90	< 0.0001	0.84	< 0.0001	0.87	< 0.0001
			Hybrid-type IR	0.81	< 0.0001	0.82	< 0.0001	0.85	< 0.0001	0.82	< 0.0001	0.89	< 0.0001	0.83	< 0.0001	0.86	< 0.0001
		ULDCT	DLR	0.76	< 0.0001	0.78	< 0.0001	0.80	< 0.0001	0.77	< 0.0001	0.84	< 0.0001	0.78	< 0.0001	0.81	< 0.0001
			Hybrid-type IR	0.74	< 0.0001	0.76	< 0.0001	0.78	< 0.0001	0.75	< 0.0001	0.82	< 0.0001	0.76	< 0.0001	0.79	< 0.0001
0.5	512 × 512	SDCT	DLR	0.85	< 0.0001	0.86	< 0.0001	0.89	< 0.0001	0.85	< 0.0001	0.93	< 0.0001	0.87	< 0.0001	0.90	< 0.0001
			Hybrid-type IR	0.84	< 0.0001	0.85	< 0.0001	0.88	< 0.0001	0.84	< 0.0001	0.92	< 0.0001	0.86	< 0.0001	0.89	< 0.0001
		RDCT	DLR	0.84	< 0.0001	0.85	< 0.0001	0.88	< 0.0001	0.84	< 0.0001	0.92	< 0.0001	0.86	< 0.0001	0.89	< 0.0001
			Hybrid-type IR	0.82	< 0.0001	0.83	< 0.0001	0.86	< 0.0001	0.83	< 0.0001	0.90	< 0.0001	0.84	< 0.0001	0.87	< 0.0001
		ULDCT	DLR	0.75	< 0.0001	0.77	< 0.0001	0.79	< 0.0001	0.76	< 0.0001	0.83	< 0.0001	0.77	< 0.0001	0.80	< 0.0001
			Hybrid-type IR	0.76	< 0.0001	0.78	< 0.0001	0.80	< 0.0001	0.77	< 0.0001	0.84	< 0.0001	0.78	< 0.0001	0.81	< 0.0001
0.25	512 × 512	SDCT	DLR	0.85	< 0.0001	0.86	< 0.0001	0.89	< 0.0001	0.85	< 0.0001	0.93	< 0.0001	0.87	< 0.0001	0.90	< 0.0001
			Hybrid-type IR	0.83	< 0.0001	0.84	< 0.0001	0.87	< 0.0001	0.84	< 0.0001	0.91	< 0.0001	0.85	< 0.0001	0.88	< 0.0001
		RDCT	DLR	0.83	< 0.0001	0.84	< 0.0001	0.87	< 0.0001	0.84	< 0.0001	0.91	< 0.0001	0.85	< 0.0001	0.88	< 0.0001
			Hybrid-type IR	0.82	< 0.0001	0.83	< 0.0001	0.86	< 0.0001	0.83	< 0.0001	0.90	< 0.0001	0.84	< 0.0001	0.87	< 0.0001
		ULDCT	DLR	0.75	< 0.0001	0.77	< 0.0001	0.79	< 0.0001	0.76	< 0.0001	0.83	< 0.0001	0.77	< 0.0001	0.80	< 0.0001
			Hybrid-type IR	0.72	< 0.0001	0.74	< 0.0001	0.76	< 0.0001	0.73	< 0.0001	0.80	< 0.0001	0.74	< 0.0001	0.77	< 0.0001
0.25	1024 × 1024	SDCT	DLR	0.83	< 0.0001	0.84	< 0.0001	0.87	< 0.0001	0.84	< 0.0001	0.91	< 0.0001	0.85	< 0.0001	0.88	< 0.0001
			Hybrid-type IR	0.82	< 0.0001	0.83	< 0.0001	0.86	< 0.0001	0.83	< 0.0001	0.90	< 0.0001	0.84	< 0.0001	0.87	< 0.0001
		RDCT	DLR	0.82	< 0.0001	0.83	< 0.0001	0.86	< 0.0001	0.83	< 0.0001	0.90	< 0.0001	0.84	< 0.0001	0.87	< 0.0001
			Hybrid-type IR	0.80	< 0.0001	0.81	< 0.0001	0.84	< 0.0001	0.81	< 0.0001	0.88	< 0.0001	0.82	< 0.0001	0.85	< 0.0001
		ULDCT	DLR	0.74	< 0.0001	0.76	< 0.0001	0.78	< 0.0001	0.75	< 0.0001	0.82	< 0.0001	0.76	< 0.0001	0.79	< 0.0001
			Hybrid-type IR	0.73	< 0.0001	0.75	< 0.0001	0.77	< 0.0001	0.74	< 0.0001	0.81	< 0.0001	0.75	< 0.0001	0.78	< 0.0001

*SDCT* standard-dose CT, *RDCT* reduced-dose CT, *ULDCT* ultra-low-dose CT, *IR* iterative reconstruction, *DLR* deep learning reconstruction

Results of ROC analyses for all lung texture evaluations for all CT protocols are shown in Table 6. Assessments of all lung radiological findings except for areas of consolidation and nodules or masses showed that areas under the curve (AUCs) of ULDCT reconstructed with hybrid-type IR, which were reconstructed for each section thickness with a 512 × 512 or 1024 × 1024 matrix, (0.91 ≤ AUC ≤ 0.97) were significantly smaller than those reconstructed with DLR (0.97 ≤ AUC ≤ 1, *p* < 0.05) and the standard protocol (0.98 ≤ AUC ≤ 1, *p* < 0.05). For emphysema evaluation, values of AUCs of RDCTs reconstructed with DLR and hybrid-type IR at 1-mm section thickness, (DLR: AUC = 0.97, hybrid-type IR: AUC = 0.97), 0.5-mm section thickness, (DLR: AUC = 0.97, hybrid-type IR: AUC = 0.97) and 0.25-mm section thickness with 512 × 512 and 1024 × 1024 matrixes (each DLR: AUC = 0.97, each hybrid-type IR: AUC = 0.97) were significantly smaller than the value for the standard protocol (*p* < 0.05). Moreover, assessment of honeycombing showed that values of AUCs of ULDCT reconstructed with DLR at all section thicknesses with 512 × 512 or 1024 × 1024 matrixes (AUC = 0.99) were significantly smaller than for the standard protocol (*p* < 0.05).

**Table 6:** ROC analyses of all lung texture evaluations for all CT protocols at a given section thickness

Section thickness (mm)	Reconstruction matrix	HDCT protocols	Reconstruction method	Emphysema	GGO	Reticulation	Nodular lesions	Consolidation	Honeycombing	Nodules or Mass
				AUC	AUC	AUC	AUC	AUC	AUC	AUC
1	512 × 512	SDCT	DLR	0.99	0.99	0.98	0.99	1	1	0.99
			Hybrid-type IR	0.99	0.99	0.98	0.99	1	1	0.99
		RDCT	DLR	0.97^+^	0.99	0.98	0.99	1	1	0.99
			Hybrid-type IR	0.97^+^	0.99	0.98	0.99	1	1	0.99
		ULDCT	DLR	0.97^+^	0.99	0.98	0.99	1	0.99^+^	0.99
			Hybrid-type IR	0.92*^, +^	0.95*^, +^	0.91*^, +^	0.96*^, +^	1	0.97*^, +^	0.99
0.5	512 × 512	SDCT	DLR	0.99	0.99	0.98	0.99	1	1	0.99
			Hybrid-type IR	0.99	0.99	0.98	0.99	1	1	0.99
		RDCT	DLR	0.97^+^	0.99	0.98	0.99	1	1	0.99
			Hybrid-type IR	0.97^+^	0.99	0.98	0.99	1	1	0.99
		ULDCT	DLR	0.97^+^	0.99	0.98	0.99	1	0.99^+^	0.99
			Hybrid-type IR	0.92*^, +^	0.95*^, +^	0.91*^, +^	0.96*^, +^	1	0.97*^, +^	0.99
0.25	512 × 512	SDCT	DLR	0.99	0.99	0.98	0.99	1	1	0.99
			Hybrid-type IR	0.99	0.99	0.98	0.99	1	1	0.99
		RDCT	DLR	0.97^+^	0.99	0.98	0.99	1	1	0.99
			Hybrid-type IR	0.97^+^	0.99	0.98	0.99	1	1	0.99
		ULDCT	DLR	0.97^+^	0.99	0.98	0.99	1	0.99^+^	0.99
			Hybrid-type IR	0.92*^, +^	0.92*^, +^	0.91*^, +^	0.96*^, +^	1	0.91*^, +^	0.99
0.25	1024 × 1024	SDCT	DLR	0.99	0.99	0.98	0.99	1	1	0.99
			Hybrid-type IR	0.99	0.99	0.98	0.99	1	1	0.99
		RDCT	DLR	0.97^+^	0.99	0.98	0.99	1	1	0.99
			Hybrid-type IR	0.97^+^	0.99	0.98	0.99	1	1	0.99
		ULDCT	DLR	0.97^+^	0.99	0.98	0.99	1	0.99^+^	0.99
			Hybrid-type IR	0.92*^, +^	0.92*^, +^	0.91*^, +^	0.96*^, +^	1	0.91*^, +^	0.99

*SDCT* standard-dose CT, *RDCT* reduced-dose CT, *ULDCT* ultra-low-dose CT, *IR* iterative reconstruction, *DLR* deep learning reconstruction

*Significant difference with CT obtained at the same radiation dose and reconstructed with DLR for the same section thickness (*p* < 0.05)

^+^Significant difference with standard-dose CT reconstructed with hybrid-type IR at 1-mm section thickness (i.e., standard protocol) (*p* < 0.05)

## Discussion

Our results firstly suggest that DLR is more effective than hybrid-type IR for radiation dose reduction for chest HDCT at each section thickness with 512 × 512 or 1024 × 1024 matrixes. Moreover, it was found that DLR, as compared with hybrid-type IR, could significantly improve image noise and SNR for lung parenchyma and detection accuracies of all lung textures except for areas of consolidation, nodules or masses on ULDCT at each section thickness with 512 × 512 or 1024 × 1024 matrixes. To the best of our knowledge, no reports have been published on the efficacy of DLR as compared with that of hybrid-type IR for the seven lung texture evaluations assessed in this study and based on the glossary terms for thoracic imaging published by the Fleischner Society for HDCT with standard, reduced and ultra-low-dose levels at each section thickness with 512 × 512 or 1024 × 1024 matrixes.

A comparison of DLR and hybrid-type IR for quantitative and qualitative image qualities showed that the use of DLR was significantly improve each image quality on HDCT at each radiation dose level and section thickness with 512 × 512 or 1024 × 1024 matrixes. Also, RDCT reconstructed with hybrid-type IR and ULDCT reconstructed with DLR and hybrid-type IR were significantly inferior to SDCT reconstructed with hybrid-type IR. Furthermore, these qualitative results were reproducible because interobserver agreements on each index for all CT data were substantial or almost perfect. When assessed each section thickness with 512 × 512 or 1024 × 1024 matrixes, assessment of all radiological findings, except for areas of consolidation and nodules or masses, demonstrated that ULDCT reconstructed with hybrid-type IR showed significantly inferior results than did ULDCT reconstructed with DLR and the standard protocol. On the other hand, detection accuracies for emphysema evaluation on RDCTs reconstructed with DLR and hybrid-type IR were significantly inferior to that for the standard protocol. Moreover, honeycombing on ULDCT reconstructed with DLR was significantly less than that for reconstruction with the standard protocol. Also, detection accuracy for each lung texture was not significantly different for DLR and hybrid-type IR used for SDCT and RDCT. Interobserver agreements for all CT protocols in terms of accuracy of detection and of evaluation for each lung texture were determined to be substantial or almost perfect. This means that our results for all lung texture evaluations on each protocol were reproducible [27].

DLR has for image noise reduction for CT or MRI has been made available by a few vendors and has been clinically tested for various clinical purposes in the last several years [8–13]. In contrast to hybrid-type IR or MBIR, which involve a trade-off between spatial resolution and noise reduction [1–7], DLR applied to a task-based model was found to reduce image noise and increase spatial resolution simultaneously [8, 11, 13, 28]. In addition, DLR was trained by using a deep convolutional neural network (DCNN) with a pair of low- and high-quality CT images. The low-quality image was obtained with low radiation doses and reconstructed with hybrid IR, and the high-quality image was acquired with routine doses and reconstructed with a customized model-based IR algorithm [8, 11, 13, 28]. Our results were therefore as expected and fully compatible with those of previous studies [8–13]. DLR can thus be considered more effective for radiation dose reduction while maintaining accuracy of lung texture detection except for emphysema on ULDCT, although DLR can improve image quality of CT without improving detection accuracy for every lung texture on SDCT and RDCT when used in routine clinical practice.

There are a few limitations to this study. First, the study population was limited and HDCT as was used for reconstruction with hybrid-type IR and DLR obtained from a single vendor. In addition, hybrid-type IR and DLR were used at the standard level, although there are other applicable levels of hybrid-type IR or DLR methods. Moreover, only three levels of radiation dose reduction were used, while other dose levels for HDCT were not tested. Our study results were thus affected by these factors, so that further investigations would be warranted to determine the actual significance of HDCT as well as DLR in routine clinical practice. Second, it has been suggested in the literature that AEC is effective for radiation dose reduction [29–33]]. Although AEC has been utilized in routine clinical practice, we did not use this technique for radiation dose reduction. The use of AEC for CT scanners essentially provides programmed dynamic adjustment of the tube current to achieve consistent image quality among patients and for a single patient. This means that further radiation dose reduction can be achieved by using AEC for RDCT and ULDCT examinations, so that a study using a combination of AEC, DLR and hybrid-type IR is clearly warranted. Third, the slice level used for quantitative and qualitative assessments of each CT protocol in this study were different because each radiological finding was presented at different slice levels. Therefore, this fact may be affected to our study results.

In conclusion, DLR has is potentially more effective than hybrid-type IR for image quality improvement and lung texture evaluation on standard-, reduced- and ultra-low-dose CTs obtained at HDCT with an UHR-CT system and reconstructed at 0.25-mm, 0.5-mm and 1-mm section thicknesses with 512 × 512 or 1024 × 1024 matrixes for patients with a variety of pulmonary diseases.

## Abbreviations

MDCT: Multidetector row CT
IR: Iterative reconstruction
DLR: Deep learning reconstruction
HDCT: High-definition CT
UHR-CT: Ultra-high-resolution CT
SHR-CT: Super-high-resolution CT
NR: Normal resolution
HR: High resolution
SHR: Super-high-resolution
SNR: Signal-to-noise ratio
CNR: Contrast-to-noise ratio
CTDI_vol_: CT dose index volume
SDCT: Standard-dose CT
RDCT: Reduced-dose CT
ULDCT: Ultra-low-dose CT
COPD: Chronic obstructive pulmonary disease
ILD: Interstitial lung disease
GOLD: Global Initiative for Chronic Obstructive Lung Disease
IPF: Idiopathic pulmonary fibrosis
MIA: Minimally invasive adenocarcinoma
NTM: Nontuberculous mycobacteria
AIS: Adenocarcinoma in situ
NSIP: Non-specific interstitial pneumonia
PSS: Progressive systemic sclerosis
COVID-19: Coronavirus disease 2019
ROC: Receiver operating characteristic
GGO: Ground glass opacity
AEC: Automatic exposure control
MCTD: Mixed connective tissue disease
ROI: Region of interest
